# Synergistic Effect of Brassinosteroid and Jasmine Extract on Promoting Rice Ratooning Ability

**DOI:** 10.3390/plants15132090

**Published:** 2026-07-05

**Authors:** Long Zhang, Qiang Cai, Yan Gan, Hang Yu, Shiyong Cui, Panyu Zhao, Shuxin Zhang, Kailing Xiao, Chenran Chen, Wenfang Lin, Wenxiong Lin, Wenfei Wang, Xuelian Yang

**Affiliations:** 1 College of Life Sciences, Fujian Agriculture and Forestry University, Fuzhou 350002, China; 18994008036@163.com (L.Z.); 12427025029@fafu.edu.cn (Q.C.); 13686795309@163.com (Y.G.); yuhangdaisyu@gmail.com (H.Y.); 18354774693@163.com (S.C.); panyu_zhao@outlook.com (P.Z.); zhangshuxin0310@163.com (S.Z.); 18059472575@163.com (K.X.); linwf@fafu.edu.cn (W.L.); wenfeiwang@fafu.edu.cn (W.W.); 2 Fujian Provincial Key Laboratory of Agroecological Processing and Safety Monitoring, Fujian Agriculture and Forestry University, Fuzhou 350002, China; lwx@fafu.edu.cn; 3 Key Laboratory of Crop Ecology and Molecular Physiology, Fujian Agriculture and Forestry University, Fuzhou 350002, China; 4 Luoyuan Association for Science and Technology, Fuzhou 350600, China; 13696832058@139.com

**Keywords:** ratoon rice, ratooning ability, brassinosteroid, jasmine extract, ratooning enhancer

## Abstract

Ratoon rice cultivation is a significant practice for enhancing land productivity and food security. Ratooning ability is a key determinant of ratoon season crop (RC) yield and is influenced by genetic, agronomic, and hormonal factors. This study aimed to evaluate the effects of foliar-applied ratooning enhancers, formulated with plant hormones and botanical extracts, on the growth and regeneration of a *japonica*–*indica* hybrid rice cultivar, ‘Qingxiangyou 19 Xiang’. Treatments included gibberellin (GA), low, medium, and high concentrations of brassinosteroid (BR), each with or without jasmine extract (JE), alongside proline and zinc chloride (ZnCl_2_) as supporting components. These solutions were applied twice at 5 and 15 days after flowering (DAF) of the main crop (MC). The results showed that GA treatment increased plant height and panicle length but reduced MC tiller number. BR treatments did not affect plant height but significantly increased the 1000-grain weight. Crucially, while BR alone had no significant effect on ratooning ability, the BR-JE combined application, particularly at medium (MBR-JE) and high (HBR-JE) concentrations, significantly increased ratoon tiller number and enhanced ratooning ability. However, the HBR-JE combination increased grain chalkiness. In conclusion, the foliar application of BR combined with JE during the flowering stage effectively promotes ratooning ability without compromising MC yield, offering a promising agronomic strategy for sustainable ratoon rice production.

## 1. Introduction

Rice (*Oryza sativa* L.) is one of the most important food crops in the world, providing approximately 21% of the food supply for nearly half of the global population [1]. RC is a cultivation practice in which, after the harvest of the MC, dormant buds on the stubble are managed to outgrow and produce a second harvest [2]. With a documented history in China spanning over 1700 years [3], it is particularly suited to regions where thermal and light resources are insufficient for double cropping but are sufficient for a single crop followed by a ratoon. RC has also been widely adopted in other countries, including India, Japan, Philippines, Indonesia, Thailand, Vietnam, the United States, Malaysia and Nigeria [4]. By improving the utilization efficiency of farmland and light temperature resources, this model can increase total grain output and farmers’ income [5]. Notably, the RC often exhibits superior grain quality compared to the MC, including improved milling traits, higher milled and head rice rates, and better appearance with reduced chalkiness [4].

A critical trait governing the success and yield potential of RC is ratooning ability. Ratooning ability is a complex, quantitatively inherited characteristic, modulated by the interplay of genetic background, environmental conditions, and agronomic management practices [6]. The germination pattern of axillary buds varies distinctly between subspecies: in *indica* rice, active buds are predominantly located at upper nodes, whereas in *japonica* rice, they are mainly concentrated at basal nodes [7]. Recent studies demonstrated that cultivation practices profoundly influence ratooning ability. Key factors include fertilizer application [8,9,10], stubble height [11], irrigation management [12], and harvest time [13].

Beyond these agronomic measures, plant hormones play an equally pivotal role in regulating bud dormancy and outgrowth. Exogenous application of GR24, a synthetic strigolactone analog, significantly suppresses axillary bud outgrowth [14]. Proteomic profiling of axillary bud development has implicated BR-related proteins in the ratooning regulation [15]. Specifically, brassinolide stabilizes the OsBZR1 protein, promoting its interaction with D53 to form a transcriptional complex that suppresses the expression of the tillering inhibitor *FC1*, thereby positively regulating tillering [16]. By reducing a disulfide bond, ratooning ability associated protein (RRA3) inhibits dimerization of cytokinin receptor OHK4, thereby attenuating cytokinin signaling and diminishing ratooning ability [17]. GAs also contribute to this process, as foliar application of GA during different growth stages of the MC has been shown to significantly affect ratoon seedling vigor and yield formation [18]. GA promotes the degradation of DELLA protein, thereby removing the protection of SLR1 on MOC1 in rice and the inhibition of DELLA on SPL9 in *Arabidopsis*, thereby inhibiting the formation of axillary buds [19,20].

In recent years, the application of natural extracts and nutrients in enhancing crop resistance and yield has received extensive attention. Jasmine extract (JE) is rich in jasmonic acid (JA) derivatives and phenolic substances, and has potential functions in regulating plant growth and development. Exogenous application of methyl jasmonate (MeJA) enhances the antioxidant capacity of rice leaves, improves the physicochemical properties of starch, and thereby increases yield and quality under adverse stress conditions such as drought [21]. Proline, as an important compatible solute and osmotic regulatory substance in plants, plays multiple physiological roles in rice’s response to abiotic stress. Exogenous application of proline can significantly enhance rice’s salt tolerance, promote chlorophyll synthesis, and increase the activity of antioxidant enzymes, protecting membrane integrity and photosynthetic efficiency [22,23]. Furthermore, combining proline with salicylic acid (SA) or betaine can regulate stomatal movement, carbon-nitrogen metabolism, and grain filling, synergistically mitigating the negative impacts of drought and high-temperature stress on yield [24,25]. Zinc (Zn) exists in plants in the form of enzyme cofactors or structural components, and is widely involved in fundamental biochemical processes such as protein synthesis, carbohydrate metabolism, cell division, and gene expression. It is crucial for maintaining the normal physiological metabolism of plants [26]. A large number of studies have reported that the application of Zn fertilizer not only enhances the antioxidant system and photosynthesis of rice, thereby increasing yield and improving rice quality, but also can simultaneously improve the nutritional quality of rice [27,28].

Despite these advances, most studies have focused on individual hormones or nutrients, and little is known about the combined effects of BR, GA, and natural extracts such as JE applied at specific stages of the MC on subsequent ratooning ability. Furthermore, the potential synergistic role of proline and Zn in supporting bud outgrowth after harvest has not been systematically evaluated in a ratoon rice system. Based on this, the present study employed the widely cultivated *japonica*–*indica* hybrid rice cultivar ‘Qingxiangyou 19 Xiang’ as experimental material. We established two foliar spray treatments at key grain-filling stages of the MC at 5 and 15 DAF, to systematically evaluate the effects of different concentrations of GA and BR, as well as their combinations with JE, proline, and ZnCl_2_ on ratooning ability. We hypothesized that (1) BR would promote ratooning ability in a concentration-dependent manner, (2) JE would synergize with BR to further enhance bud outgrowth, and (3) the inclusion of proline and Zn would provide stress-protective effects that indirectly improve ratoon vigor. Our experiment included treatments with low, medium- and high-concentration ratoon enhancer solutions (GA, LBR, MBR, HBR) and their combinations with jasmine extract (GA-JE, LBR-JE, MBR-JE, HBR-JE), with distilled water as the control. The objective was to identify the optimal ratoon enhancer formulation, thereby providing a novel technical strategy for achieving high and stable yields in ratoon rice production.

## 2. Materials and Methods

### 2.1. Plant Materials and Growth Conditions

**Figure 1:**
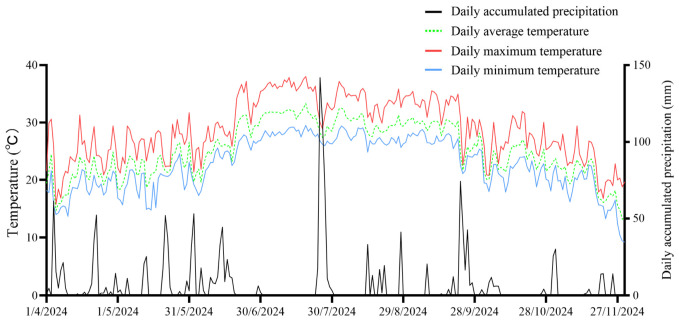
Average temperature, maximum temperature, minimum temperature, and daily accumulated precipitation during the trials in 2024.

“Qingxiangyou 19 Xiang”, a *japonica*–*indica* hybrid rice cultivar, was cultivated as ratoon rice in Luoyuan, Fuzhou City, Fujian Province (119°38′ E, 26°25′ N). Daily meteorological data, including maximum, average, and minimum temperatures and accumulated precipitation, were collected from a local weather station throughout the MC and RC (Figure 1). Rice seedlings were raised in standard plastic seedling trays (40 cm × 100 cm) filled with 3 cm thick commercial matrix soil (organic matter > 20%, pH 5.5–6.5). Seeds were sown in late April 2024, and 30-day-old seedlings were transplanted to the paddy field on 20 May 2024, at a hill spacing of 25 cm × 20 cm (20 hills per m^2^), with 1 seedling per hill. The physical and chemical properties of the soil were measured as: 1.55 g·kg^−1^ total nitrogen, 0.55 g·kg^−1^ available phosphorus, 0.51 g·kg^−1^ available potassium. Fertilizer application for the MC followed local high-yield management practices. A total of 225 kg·hm^−2^ pure nitrogen was applied with a split ratio of base fertilizer. Tillering fertilizer:booting fertilizer:grain fertilizer = 4:2:3:1. The N:P:K ratio was 1:0.5:0.8. Phosphorus was applied entirely as basal fertilizer, while potassium was split 5:5 between basal and booting stages. For the RC, 160 kg·hm^−2^ pure nitrogen was applied as a top-dressing 3 days after the MC harvest to promote ratoon bud germination and early growth. Irrigation was managed conventionally: a shallow water layer (3–5 cm) was maintained from transplanting until 10 days before harvest, at which point the field was drained to facilitate mechanical or manual harvesting. The field was re-flooded immediately after the MC harvest to promote ratoon bud germination, and a thin water layer was maintained throughout the RC until 10 days before it was harvested.

### 2.2. Experimental Design

**Figure 2:**
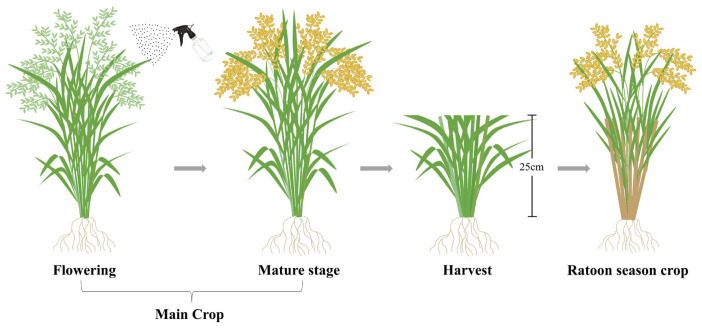
Schematic of ratooning enhancer application. Foliar spray treatments were applied to the MC panicles twice, at 5 and 15 DAF, respectively. At the mature stage of MC, the panicles were harvested, leaving a stubble height of 25 cm. Subsequently, regenerated tillers emerged and produced the RC.

Foliar spray treatments were applied to the panicles twice at 5 and 15 DAF, corresponding to 25 July and 4 August 2024 (Figure 2). The treatments comprised a series of ratooning-enhancer solutions formulated with phytohormones GA and BR at low, medium, and high concentrations, and JE, in combination with proline and ZnCl_2_, and distilled water was used as a control. The exact compositions of all treatments are provided in Table 1. Briefly, all experimental groups (including GA, GA-JE, LBR, LBR-JE, MBR, MBR-JE, HBR, and HBR-JE) contained a fixed concentration of proline (1.7 mM). The concentration of ZnCl_2_ was 27 mM in the hormone-only treatments (GA, LBR, MBR, HBR) and 20 mM in the treatments that also contained JE (GA-JE, LBR-JE, MBR-JE, HBR-JE). JE powder (Cat No. TEML060426) used in this study was a commercially available water-soluble extract powder purchased from Shaanxi Tianen Biotechnology Co., Ltd (Shaanxi, China). JE (1 g/L) was added only in the JE-combined treatments. Gibberellin (GA_3_) was used at 0.1 mM in the GA and GA-JE treatments. BR (24-epibrassinolide) was applied at 0.1 μM (LBR), 1 μM (MBR), or 10 μM (HBR), with or without JE.

Each formulation was prepared fresh on the day of application by dissolving the respective chemicals in distilled water. The pH of all solutions was adjusted to 6.0–6.5 using dilute NaOH or HCl to avoid phytotoxicity. Each formulation was applied at a volume of 500 mL per 10 plants (approximately 50 mL per plant), with three independent biological replicates per treatment (each replicate consisted of 3 plants). The spray was applied evenly to both sides of the panicles and upper leaves using a hand-held sprayer (1.5 L capacity, conical nozzle) until slight runoff was observed. Applications were conducted in the late afternoon (17:00–18:00) under windless conditions to minimize rapid evaporation and photodegradation. Care was taken to avoid drift between plots by using protective shields during spraying.

### 2.3. Phenotypic Assessment and Sampling

MC measurements. At the mature stage of MC (approximately 30 days after the second spray), phenotypic traits and yield components were quantified. The following parameters were recorded for each replicate:

- Plant height: measured from the soil surface to the tip of the tallest panicle (excluding awns) using a measuring tape (cm).
- Effective tiller number: the number of panicles per plant bearing ≥5 filled grains.
- Number of grains per panicle: counted from five representative panicles per plant and averaged.
- Seed-setting rate: calculated as (filled grains/total grains) × 100%, assessed using the water-floatation method.
- 1000-grain weight: measured using an electronic balance (0.01 g precision) after oven-drying to 13% moisture content.
- Yield per plant: total grain weight per plant, adjusted to 13% moisture content.
- Grain dimensions: grain length, width, and length/width ratio were measured on 20 randomly selected fully filled grains per replicate using a digital vernier caliper (0.01 mm precision).

RC measurements. At the maturity of the RC (approximately 65 days after MC harvest), the following parameters were recorded:

- Number of ratoon tillers: productive tillers bearing panicles per plant.
- Ratooning ability (RA): calculated as the ratio of ratoon tillers to effective main-crop tillers, expressed as a percentage: RA (%) = (RC tillers/MC tillers) × 100%.

For each treatment, three biological replicates were used, with three plants harvested and measured per replicate (i.e., 9 plants per treatment total). Plants were selected from the central rows of each plot to avoid border effects.

### 2.4. Statistical Analysis

All data were analyzed and plotted with GraphPad Prism 8. Before analysis, normality of residuals was checked using the Shapiro–Wilk test, and homogeneity of variances was verified using Levene’s test. Since both assumptions were met, data were subjected to one-way analysis of variance (ANOVA). Statistical significance between treatment groups was determined by Fisher’s least significant difference (LSD) post hoc test at a significance level of α = 0.05. All data are presented as mean ± standard deviation (SD) (n = 3), and *p* values are indicated in the respective figure legends.

## 3. Results

### 3.1. Effects of Ratooning Enhancers on Rice Agronomic Traits of MC

To assess the effects on RA, different ratooning enhancer formulations were applied as foliar sprays during the MC grain-filling stage of the *japonica*–*indica* hybrid rice cultivar ‘Qingxiangyou 19 Xiang’ (Figure 2). The tested ratooning enhancers consisted of phytohormones, water-extracted JE, in combination with consistent supporting components such as proline and ZnCl_2_. Proline was included for its ability to enhance plant vigor and stress tolerance, thereby indirectly supporting tillering potential [29], while Zn was selected for its role in improving stress and tiller recovery via hormonal modulation in tiller buds [30]. Two growth-promoting hormones, GA and BR, were selected based on their reported roles in regulating axillary bud development. GA has been shown to inhibit tillering bud growth [19], whereas BR promotes the outgrowth of axillary buds [15,16]. JE was also included in the ratooning enhancer application, as JA is generally considered to be induced in plants by cutting treatment.

**Figure 3:**
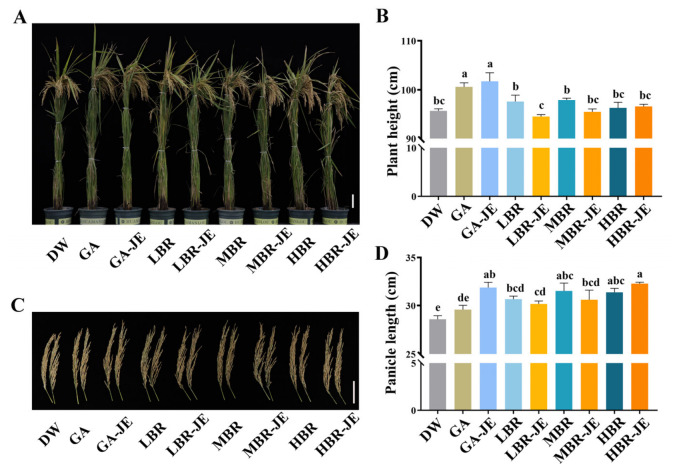
Phenotypic effects of different ratooning enhancers on rice plant morphology. MC was treated with ratooning-enhancer solutions twice at the grain-filling stage, and agronomic traits were observed and measured at the mature stage. (**A**) Whole plant phenotypes at mature stage of MC. (**B**) The statistical analysis of plant height. (**C**) Panicle phenotypes. (**D**) Panicle length. Scale bar = 10 cm in (**A**,**B**). Data represent mean ± SD (n = 3). Different letters indicate significant differences among treatments (*p* < 0.05) based on one-way ANOVA and LSD multiple range test.

During the grain-filling stage, at 5 and 15 DAF, different ratooning enhancers were sprayed twice onto the rice plants (Figure 2). The nine experimental treatments included: 0.1 mM GA_3_ (GA), a mixture of GA_3_ and JE (GA-JE), three concentrations of BR (LBR: 0.1 μM, MBR: 1 μM, HBR: 10 μM), and corresponding BR-JE mixtures (LBR-JE, MBR-JE, HBR-JE), with a distilled water spray serving as the control (DW) (Table 1). Compared to the control, GA treatment significantly increased plant height, whereas BR application at any concentration had no significant effect on plant height. The addition of JE to either GA or BR also did not influence plant height relative to the hormone-only treatments (Figure 3A,B). In contrast, all treatments, GA and BR at all concentrations, and their respective combinations with JE, significantly increased panicle length compared to the control (Figure 3C,D).

**Figure 4:**
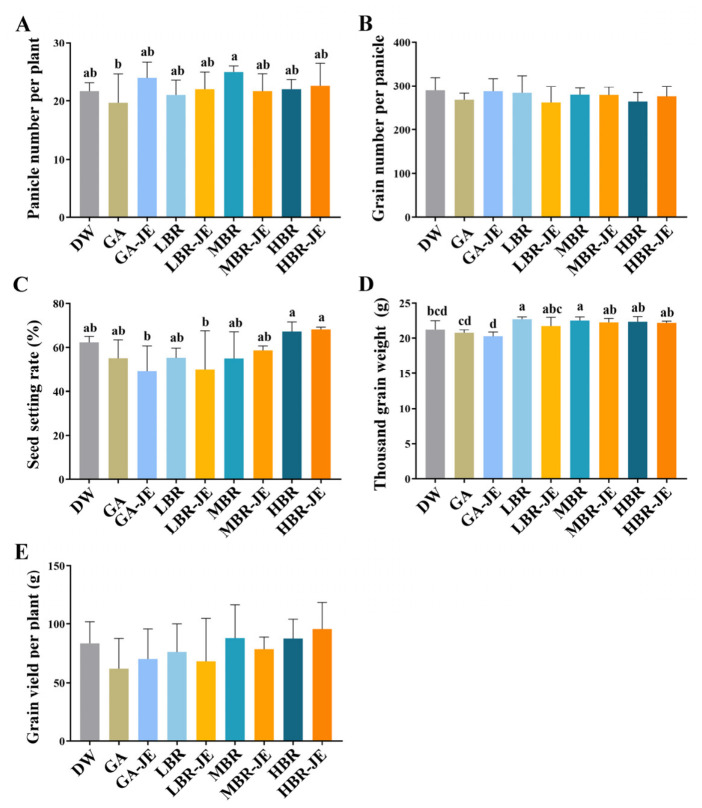
Yield performance of MC in response to different ratooning-enhancer treatments. (**A**) Effective panicle number per plant. (**B**) Grain number per panicle. (**C**) Seed setting rate. (**D**) Thousand grain weight. (**E**) Grain yield per plant. Data represent mean ± SD (n = 3). Different letters indicate significant differences among treatments (*p* < 0.05) based on one-way ANOVA and LSD multiple range test.

The yield and yield components of the MC under different treatments were evaluated. GA treatment slightly reduced the number of effective panicles per plant and the 1000-grain weight compared to the control (Figure 4A,D). Among the three BR concentrations tested, MBR produced the highest number of effective panicles (Figure 4A), while HBR resulted in the highest seed setting rate (Figure 4C). All three BR gradients greatly increased 1000-grain weight, while they did not affect grain numbers per panicle (Figure 4B,D). The addition of JE did not significantly alter any yield component across the treatments (Figure 4). Consequently, neither GA, BR, nor JE significantly influenced the per-plant yield of MC (Figure 4E). In summary, the ratooning enhancers slightly modified plant morphology and yield components but without significantly affecting the final MC yield.

### 3.2. Effects of Ratooning Enhancers on Rice Grain Quality of MC

**Figure 5:**
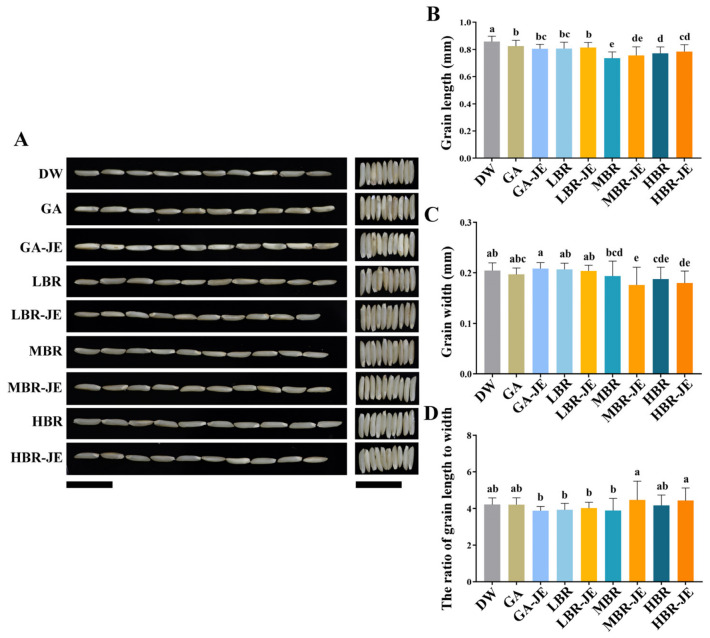
Grain appearance quality of MC after treating with different ratooning enhancers. (**A**) Dehulled grain of MC. (**B**) Grain length. (**C**) Grain width. (**D**) Ratio of grain length to width. Data represent mean ± SD (n = 10). Scale bar = 1 cm in (**A**). Different letters indicate significant differences among treatments (*p* < 0.05) based on one-way ANOVA and LSD multiple range test.

To evaluate the effects of different ratooning enhancer treatments on grain quality, key quality traits of the MC grains were measured. All the BR and BR-JE treatments slightly reduced the chalk rice rate. Grain length analysis revealed that treatments with GA, GA-JE or BR showed no significant differences compared to the DW control (Figure 5A,B). Notably, the combination of BR and JE, especially LBR-JE and HBR-JE reduced the grain length (Figure 5A,B). In contrast to length, grain width remained relatively stable across all treatments, with no significant differences observed among treatments (Figure 5C). The ratio of grain length to width was also calculated. GA and GA-JE treatments increased the ratio, while LBR-JE and HBR-JE reduced the ratio (Figure 5D). Overall, BR combined with JE effectively modulated grain appearance by reducing grain length and the length-to-width ratio, while slightly improving grain quality by reducing chalkiness.

### 3.3. Effects of Ratooning Enhancers on Ratoon Ability

**Figure 6:**
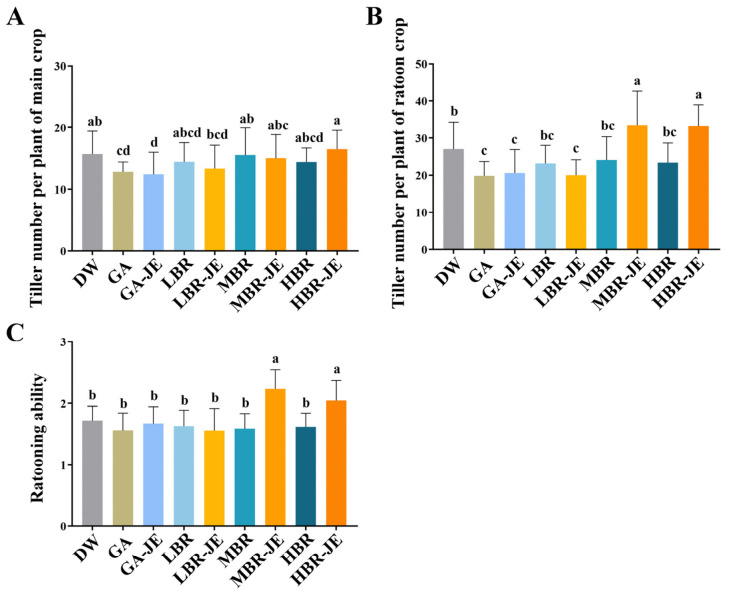
Effects of different ratooning enhancers on ratooning ability. (**A**) Tiller number per plant in MC. (**B**) Tiller number per plant in RR. (**C**) Ratooning ability (ratio of ratoon tillers to MC tillers). Data represent mean ± SD (n = 12). Different letters indicate significant differences among treatments (*p* < 0.05) based on one-way ANOVA and LSD multiple range test.

To assess the impact of different treatments on rice ratooning, tiller numbers of both MC and RR plants were measured. Compared to the control, GA and GA-JE treatments significantly reduced MC tiller numbers, whereas BR and BR-JE treatments showed no significant effects (Figure 6A). In the ratoon season, tiller numbers were dramatically reduced after GA and GA-JE treatments. In contrast, BR treatments alone showed no significant effect on tiller numbers of RR (Figure 6B). However, when combined with JE, the effect varied by BR concentration: RR tiller numbers decreased in the LBR-JE group but increased dramatically by 20.1% and 17.5% in both the MBR-JE and HBR-JE groups. Ratooning ability, calculated as the ratio of RR to MC tiller numbers, was substantially improved in the MBR-JE and HBR-JE treatments and respectively improved ratooning ability by 31.4% and 20.5% (Figure 6C). These results indicate that the combined application of BR and JE, particularly at medium and high concentrations of BR, effectively enhances ratooning ability in rice.

## 4. Discussion

### 4.1. Effects of Hormones and JE on Agronomic Traits

Plant hormones are endogenous signaling molecules that, despite their extremely low concentrations, play essential roles in regulating plant growth and development [31]. Our findings align with the established functions of GA and BR. The application of GA significantly increased plant height and panicle length but slightly reduced the number of effective panicles in the MC (Figure 3 and Figure 4). This is consistent with previous reports that exogenous GA primarily promotes internode elongation, often at the expense of tiller development, leading to a decrease in productive panicles [19,32,33]. Notably, none of the treatments, including GA, caused a significant change in the final per-plant yield of the MC. This suggests that a change in a single growth parameter was compensated for by other components, possibly due to the concentration, the way or the growth stage of the hormone treatment.

In contrast, BR treatments did not affect plant height but significantly increased the 1000-grain weight (Figure 3 and Figure 4). Medium-concentration BR (MBR) produced the highest number of effective panicles, while high-concentration BR (HBR) resulted in the best seed setting rate, which is consistent with the previous studies [16]. Furthermore, the addition of JE to either hormone did not significantly modify their effects on MC yield or plant morphology (Figure 3 and Figure 4), indicating that JE’s primary role is not in modulating MC agronomic performance under these conditions. However, since our experiments had limited biological repeats and sample size, some traits showed considerable variation among treatment groups; a larger sample size will be introduced into our future experiments.

### 4.2. Effects of Enhancers on Grain Quality

Beyond yield, grain quality is a critical trait. Among plant hormones, auxin, cytokinin, and GA typically increase grain chalkiness, whereas BR uniquely reduces it [34,35]. Field studies confirm that foliar application of brassinolide can improve rice quality by significantly reducing chalkiness [36]. In our study, BR alone did not significantly affect chalkiness. However, the combination of HBR and JE significantly increased it (Figure 5), indicating that JE may interfere with or modulate BR’s role in regulating chalkiness formation. The underlying mechanism remains unclear but may be related to altered grain-filling dynamics, particularly sucrose transport and starch synthesis, under the influence of high-concentration hormones. It was reported that exogenous proline application at the initial heading stage significantly reduced chalky grain rate and chalkiness degree by regulating carbon-nitrogen metabolism, but did not notably alter grain length-to-width ratio [37], indicating that the proline may contribute to the reduction in chalkiness when co-treated with BR and JE.

### 4.3. Effects of Enhancers on Ratooning Ability

Tillering is a key determinant of rice grain yield and is intricately regulated by hormonal networks. Ratooning ability, a specific form of tiller regeneration, is governed by a complex interplay of genetic, hormonal, and environmental factors [38]. GA signal triggers the proteasomal degradation of SLR1, which leads to the degradation of MOC1, thereby inhibiting the formation and growth of rice buds [19]. Consistently, in our study, while the GA application suppressed tiller number in both the main and ratoon crops, it did not significantly alter the calculated ratooning ability (Figure 6). Additionally, the addition of stable components such as proline and Zn may indirectly support the regenerative potential of axillary buds by enhancing the overall stress resistance and hormone signal transduction efficiency of the plant [39,40,41,42,43]. In 2025, it was reported that 24-epibrassinosteroid and jasmine oil improve vegetative growth and productivity of flame seedless grapevines under abiotic stresses [44]. Water-extracted JE was tested in our enhancers, as JA is generally considered to be induced in plants by cutting treatment. JA substances, as key signaling molecules for plant defense and development, are rapidly induced to accumulate after mechanical damage (such as harvesting) [45,46]. However, few studies have reported on the biological function of water-extracted JE on plants, especially on ratoon rice.

Mechanistically, BR promotes axillary bud outgrowth, potentially by upregulating cytokinin synthesis. The transgenic rice plants with enhanced BR signaling, specifically *OsGSK2-RNAi* and *OsBZR1-OE*, all displayed the phenotype of multiple tillers [16]. In 2020, Xu et al. conducted proteomic analysis on the axillary buds and identified that multiple protein levels changed during the development of axillary buds in ratoon rice and initially revealed that the BR might be involved in regulating ratooning ability [15]. Our core finding is the synergistic effect of BR and JE in enhancing this trait, while BR applied alone also had no significant effect. Strikingly, the combination of JE with medium or high concentrations of BR significantly increased ratoon-crop tiller numbers (Figure 6B,C). In this study, applying exogenous JE at the flowering stage may pre-activate the JA signaling pathway in axillary buds, making them more prone to respond to growth-promoting signals after the MC harvest. Therefore, the BR-JE combined treatment is not a simple additive effect, but may involve cross-talk between the hormone signaling network, which ultimately promotes the release and outgrowth of axillary buds.

### 4.4. Future Research Directions and Potential Applications

Despite the promising results, several limitations should be acknowledged. First, a major limitation of this study is the use of a single rice hybrid (Qingxiangyou 19 Xiang). Ratooning ability is a quantitatively inherited trait that varies substantially across genotypes, with distinct bud emergence patterns between *indica* and *japonica* subspecies [7], and differential hormonal sensitivities widely documented in tillering regulation [16,19]. Consequently, the BR-JE synergy observed here cannot be assumed to occur uniformly across all rice cultivars. We emphasize that the present findings serve as a proof-of-concept demonstration, providing a basis for subsequent genotype-screening studies. Future investigations should systematically include multiple varieties representing diverse genetic backgrounds, including inbred and hybrid rice with contrasting ratooning abilities, to evaluate the genotype-dependency of the formulation efficacy and to identify potential interactions between treatment response and genetic factors. Second, we did not quantify dynamic changes in endogenous phytohormones within axillary buds. Therefore, the proposed hormonal cross-talk mechanism remains correlative rather than causal. Third, in this study, the JE powder used was a crude extract, and its specific active components and contents need to be further identified. This limitation restricts our ability to deeply analyze the synergistic mechanism between it and BR at the molecular level. Fourth, in our spraying experiment, we did not include a “proline + Zn alone” treatment or a “hormone alone (without proline + Zn)” or JE alone treatment as controls, since our initial objective was to investigate the effects of spraying rice with JE in combination with either GA or different concentrations of BR. We will improve our design in future studies.

To address these gaps, future investigations should pursue a multi-scale approach. First, integrating transcriptomic and metabolomic profiling by BR-JE treatment is imperative to elucidate the core genes and metabolic pathways governing this synergy. Second, multilocation field trials across diverse ecological regions and different genotypes are required to validate the broad-spectrum efficacy of the formulation. Third, refining application timing will be crucial to identifying the optimal window for maximizing yield.

Beyond addressing these knowledge gaps, the BR-JE formulation presented here holds considerable promise for sustainable agriculture. It can be directly adopted as a foliar spray in ratoon rice production systems, especially in regions where labor costs for double cropping are high but single cropping leaves thermal resources underutilized. Second, the concept of using a natural plant extract JE combined with a low-concentration phytohormone BR, which is already broadly used in rice planting, aligns with the growing global demand for green agricultural technologies and reduced chemical residues. Moreover, the same principle that pre-activating jasmonate signaling before harvesting to prime regenerative growth, might be extended to other ratoonable or perennial crops such as tea trees, sugarcane, sorghum, forage grasses, and certain vegetable crops. This study provides a proof-of-concept that combining hormone- and defense-signaling pathways can unlock hidden yield potential in ratoon and perennial cropping systems, opening new avenues for “signal priming” strategies in sustainable agriculture.

## 5. Conclusions

In conclusion, this study demonstrated that foliar application of BR combined with JE at the grain-filling stage (5 and 15 DAF) significantly enhances ratooning ability of the *japonica*–*indica* hybrid rice cultivar ‘Qingxiangyou 19 Xiang’. Specifically, the combination of JE with medium (1 µM) and high (10 µM) concentrations of BR increased ratoon tiller numbers by 20.1% and 17.5%, and improved ratooning ability by 31.4% and 20.5%, respectively, compared with the control treatment. Notably, the MBR-JE formulation achieved this enhancement without compromising MC yield or grain chalkiness, making it a promising candidate for practical field application. Our work provides new insights into the underlying mechanisms of tiller outgrowth and ratooning ability, as well as the potential roles of BR and JE in these processes, thereby laying a foundation for future studies at the physiological and molecular levels. Overall, this study presents a novel and effective agronomic strategy for ratoon rice, which will benefit future breeding programs and cultivation management.

## Figures and Tables

**Table 1:** Components of Nine Ratooning Enhancer Formulations.

	Treatment	GA_3_ (mM)	BR (μM)	Proline (mM)	ZnCl_2_ (mM)	JE (g/L)
Control group	DW	0	0	0	0	0
Experimental group	GA	0.1	0	1.7	27	0
	GA-JE	0.1	0	1.7	20	1
	LBR	0	0.1	1.7	27	0
	LBR-JE	0	0.1	1.7	20	1
	MBR	0	1	1.7	27	0
	MBR-JE	0	1	1.7	20	1
	HBR	0	10	1.7	27	0
	HBR-JE	0	10	1.7	20	1

Abbreviations: DW, distilled water. GA, GA_3_ with proline and ZnCl_2_. GA-JE, GA_3_ with jasmine extract, proline and ZnCl_2_. LBR, low concentration BR (0.1 μM) with proline and ZnCl_2_. MBR, medium concentration BR (1 μM) with proline and ZnCl_2_. HBR, high concentration BR (10 μM) with proline and ZnCl_2_.

## Data Availability

The original contributions presented in this study are included in the article. Further inquiries can be directed to the corresponding author.
